# Assessment of candidemia-attributable mortality in critically ill patients using propensity score matching analysis

**DOI:** 10.1186/cc11388

**Published:** 2012-06-14

**Authors:** Francisco J González de Molina, Cristóbal León, Sergio Ruiz-Santana, Pedro Saavedra

**Affiliations:** 1 Intensive Care Department, Hospital Universitari Mútua Terrassa, Universitat de Barcelona, Plaça Dr. Robert, (08221) Terrassa, Barcelona. Spain; 2 Intensive Care Unit, Hospital Universitario de Valme, Universidad de Sevilla, Carretera de Cádiz s/n, (41014) Seville, Spain; 3 Intensive Care Unit, Hospital Universitario Dr. Negrín, Universidad de las Palmas de Gran Canaria, Barranco de la Ballena s/n (35010) Las Palmas de Gran Canarias. Spain; 4 Mathematics Department, Universidad de las Palmas de Gran Canaria Campus Universitario de Tafira (35017), Las Palmas de Gran Canaria. Spain

## Abstract

**Introduction:**

Candidemia in critically ill patients is usually a severe and life-threatening condition with a high crude mortality. Very few studies have focused on the impact of candidemia on ICU patient outcome and attributable mortality still remains controversial. This study was carried out to determine the attributable mortality of ICU-acquired candidemia in critically ill patients using propensity score matching analysis.

**Methods:**

A prospective observational study was conducted of all consecutive non-neutropenic adult patients admitted for at least seven days to 36 ICUs in Spain, France, and Argentina between April 2006 and June 2007. The probability of developing candidemia was estimated using a multivariate logistic regression model. Each patient with ICU-acquired candidemia was matched with two control patients with the nearest available Mahalanobis metric matching within the calipers defined by the propensity score. Standardized differences tests (SDT) for each variable before and after matching were calculated. Attributable mortality was determined by a modified Poisson regression model adjusted by those variables that still presented certain misalignments defined as a SDT > 10%.

**Results:**

Thirty-eight candidemias were diagnosed in 1,107 patients (34.3 episodes/1,000 ICU patients). Patients with and without candidemia had an ICU crude mortality of 52.6% versus 20.6% (*P *< 0.001) and a crude hospital mortality of 55.3% versus 29.6% (*P *= 0.01), respectively. In the propensity matched analysis, the corresponding figures were 51.4% versus 37.1% (*P *= 0.222) and 54.3% versus 50% (*P *= 0.680). After controlling residual confusion by the Poisson regression model, the relative risk (RR) of ICU- and hospital-attributable mortality from candidemia was RR 1.298 (95% confidence interval (CI) 0.88 to 1.98) and RR 1.096 (95% CI 0.68 to 1.69), respectively.

**Conclusions:**

ICU-acquired candidemia in critically ill patients is not associated with an increase in either ICU or hospital mortality.

## Introduction

*Candida *spp. are the fourth most common cause of bloodstream infection in hospitalized patients [1-4] and a leading cause of invasive fungal infection in the ICU setting [1]. Candidemia in critically ill patients is usually a severe and life-threatening condition with a high crude mortality ranging between 35% and 75% [5,6]. In contrast, candidemia-attributable mortality is unclear, with studies showing high rates of 14.5% to 49% and others failing to demonstrate any significant increase in mortality [7].

Infection caused by *Candida *spp. develops in patients with multiple risk factors and severe underlying diseases, so that it is difficult to distinguish mortality attributed to candidemia from mortality related to the underlying illness. Thus, there is an interest in conducting case-control or matched cohort studies in which attributable mortality is obtained after matching and adjusting for confounding variables. Despite these techniques it seems almost impossible to match all factors (observable and unobservable) than can potentially have an influence upon mortality. This obstacle was overcome by Rosenbaum and Rubin [8] in 1983 who suggested matching 'cases' and 'controls' solely on their 'propensity score' - the estimated probability of being a 'case' given observable characteristics. In a scenario of ICU-acquired candidemia, each patient with candidemia is matched to a patient without candidemia who is most similar in terms of being a candidemic patient, where this probability is calculated on the basis of individual characteristics. Once the two groups are formed, the average effect is estimated for each outcome by simply computing the difference in means between the two groups. In recent years, the use of propensity score analysis in observational studies has increased considerably [9].

This study was designed to determine the attributable mortality of ICU-acquired candidemia in non-neutropenic critically ill patients using propensity score matching analysis.

## Materials and methods

### Design and study population

This was a prospective, cohort, observational and multicenter study to assess candidemia-attributable mortality in non-neutropenic adult ICU patients. Patients over the age of 18 years who were admitted for at least seven days to 36 medical-surgical ICUs of 32 tertiary care hospitals in Spain, three in Argentina, and one in France between April 2006 and June 2007 were eligible. Exclusion criteria were age under 18 years, neutropenia defined as a total leukocyte count ≤500/mm^3 ^for more than three weeks, life expectancy of less than a week, pregnant women and nursing mothers, fungal infections other than those caused by *Candida *spp., patients who had *Candida *spp. isolation or were treated with antifungal drugs during the first seven days of ICU admission, and refusal to give informed consent. The study protocol was approved by the Ethics Committee of the participating centers and informed consent was obtained from patients or patient's representatives to participate in the study and publication of results.

### Data collection

For all patients during their ICU stay, screening for *Candida *colonization was performed once a week using routine samples from a digestive focus (feces and gastric or pharyngeal aspirates), urine, skin, respiratory samples and peripheral blood. Other samples from vascular catheters, wound or drainage exudates, or other infected foci could be obtained at the discretion of the attending physician. Results were considered positive in the presence of *Candida *growth in the culture medium. Candidemia was defined as at least one blood culture positive for *Candida *spp. The onset of candidemia was defined as the day when the first positive blood culture was obtained. The different *Candida *isolates were identified at the species level. All catheter tips removed were cultured in blood agar and Sabouraud Dextrose agar (Difco Laboratories, Detroit, Mich.) by the Maki roll plate technique [10]. Catheter-related candidemia required the isolation of the same *Candida *species from blood and catheter segment when the semiquantitative catheter tip culture yielded more than 15 colony-forming units (CFU) [11]. *Candida *peritonitis is defined as the isolation of *Candida *spp. in a peritoneal sample obtained by laparotomy or percutaneous puncture in patients with associated clinical findings. Time to antifungal therapy was defined as the time interval between when the first positive *Candida *blood culture was obtained and the time when antifungal therapy was initiated. We measured time to antifungal initiation in 24-hour increments and categorized these times as day 0 (0 to 24 hours), day one (24 to 48 hours), day three (48-72 hours) and so on. Due to the observational aspect of this study, the choice of antifungal agent was left to the discretion of the treating physicians. The use of fluconazole was assumed to be inappropriate if it was prescribed for fungal bloodstream infections caused by *C. Krusei *or *C. glabrata *(if it was resistant or no susceptibility testing was done). Patients were followed until discharge from the ICU, discharge from the hospital, or death. The following variables were recorded: age, sex, date of ICU admission, dates of ICU and hospital discharge, reason for ICU admission, underlying diseases, concomitant infections, previous treatment with antibiotics or corticosteroids, and risk factors. According to diagnoses at the time of ICU admission, patients were classified as surgical, trauma, or medical. Type of surgery (abdominal, non abdominal, elective, urgent) and number of major procedures performed before and during ICU stay were recorded. Medical patients undergoing major surgery during ICU stay were considered surgical patients. Underlying diseases included insulin-dependent diabetes mellitus, neurological conditions, chronic obstructive pulmonary disease (COPD), chronic liver disease, chronic renal failure and severe heart failure. Risk factors included the following: treatment with corticosteroids with a daily dose equivalent to 20 mg prednisone for at least two weeks, use of broad spectrum antibiotics or antimicrobial drugs within ten days prior to the study, use of mechanical ventilation and urinary catheter in place on the day of enrollment. Central venous catheters, arterial catheters, total parenteral nutrition, enteral nutrition and renal replacement therapy were also recorded.

The Acute Physiology and Chronic Health Evaluation (APACHE II) score [12] and the Sequential Organ Failure Assessment (SOFA) score [13] were recorded on ICU admission, once a week thereafter, and at the time of starting antifungal treatment. Sepsis, severe sepsis, and septic shock were defined according to international sepsis definitions [14].

### Statistical analysis

Categorical variables are expressed as frequencies and percentages, and continuous variables as mean and standard deviation (SD) when data followed a normal distribution, or as median and interquartile (IQR) (25th to 75th percentile) range when distribution departed from normality. Categorical variables were compared with the chi-square (χ^2^) test or the Fisher's exact probability test, the means by the Student's *t *test and the medians by the Kruskal-Wallis test. Risk factors for death among candidemic ICU patients were determined using the Cox proportional-hazard regression model. The probability of developing candidemia was estimated using a multivariate logistic regression model that incorporated demographic data, length of ICU stay, severity indexes, comorbidities, and risk factors [15]. Variables with statistical significance in univariate analysis (*P *< 0.2) and variables of clinical relevance were included in the model. We judged that estimation of missing data was not required since in 91.6% of the patients (1,014) all data were complete (three patients with candidemia had missing data). The discriminatory power of the model was evaluated by the Hosmer-Lemeshow goodness-of-fit test and calculating the area under the receiver operating characteristics (ROC) curve (AUC). The model was considered to have good discriminatory power when AUC was greater than or equal to 0.80. Each patient with ICU-acquired candidemia was matched to the two control patients with the nearest available Mahalanobis metric matching within calipers determined by the propensity score. The caliper was defined as one quarter of the standard deviation of the logit of the propensity score [16]. To determine the effectiveness of propensity score matching in controlling for differences between patients with and without candidemia, the standardized differences tests (SDT) were calculated for each variable before and after matching. The McNemar's test was used to compare crude mortality in the matched samples. Candidemia-attributable mortality was determined by a modified Poisson regression model with a robust error variance adjusted by those variables that still presented certain misalignment defined as a SDT > 10% [17-19]. Statistical analyses were performed with SPSS 15.0 (SPSS Inc., Chicago, Illinois) and SAS 9.1 software (SAS Institute, Inc, Cary, NC).

## Results

A total of 1,107 patients fulfilled the inclusion criteria and were included in the study. Thirty-eight patients (3.4%) developed candidemia, with an incidence rate of 34.3 episodes of candidemia per 1,000 ICU patients and 1.48 episodes per 1,000 days of ICU stay (95% CI 1.05 to 2.03 episodes per 1,000 patient-days). Baseline characteristics of the study population according to the presence or absence of candidemia are shown in Table 1.

**Table 1:** Characteristics of the study patients according to infection status.

	Without candidemia (number = 1,069)	With candidemia (number = 38)	*P *value	SDT
Age, years, mean (SD)	60.0 ± 17.1	59.0 ± 17.9	.706	5.7%

Male/female (%)	67.4/32.6	63.2/36.8	.583	8.8%

APACHE II score at ICU admission, mean (SD)	18.3 ± 7.0	20.1 ± 6.5	.131	-19.24%

SOFA score at ICU admission	7 (4 to 9)	8.5 (7 to 10)	.004	

ICU length of stay (days)^a ^	17 (12 to 28)	28.5 (16.5 to 53.5)	.001	

Hospital length of stay (days)^a ^	37 (23 to 59)	37 (26 to 67)	.436	

ICU mortality, number (%)	220 (20.6)	20 (52.6))	< .001	

Overall mortality, number (%)	314 (29.6)	21 (55.3)	.001	

Diagnosis on ICU admission, number (%)			.437	

Medical	523 (48.9)	16 (42.1)		-13.68%

Surgical	369 (34.5)	17 (44.7)		20.97%

Trauma	177 (16.6)	5 (13.2)		-9.55

Multifocal colonization, total, number (%)	649 (60.7)	35 (92.1)	< .001	79.58%

Clinical condition, number (%) at the time of ICU admission			< .001	

No sepsis	497 (46.6)	1 (2.6)		-118.8%

Sepsis	297 (27.9)	11 (28.9)		2.22%

Severe sepsis	108 (10.1)	11 (28.9)		48.84%

Septic shock	164 (15.4)	15 (39.5)		56.1%

Surgery, number (%)	369 (34.4)	17 (44.7)	.202	21.18%

Abdominal surgery, number (%)	205 (19.2)	12 (31.6)	.075	28.78%

Surgical procedures, number			.302	

None	625 (58.5)	20 (52.6)		-11.89%

One	316 (29.6)	10 (26.3)		-7.36%

Two or more	128 (12.0)	8 (21.1)		24.67

Secondary peritonitis, number (%)	65 (6.1)	4 (10.5)	.306	16%

**Underlying illnesses, number (%)**				

Neurologic disease	224 (21.0)	5 (13.2)	.220	-20.83%

Trauma	197 (18.4)	7 (18.4)	.999	0%

Diabetes mellitus	183 (17.1)	7 (18.4)	.836	3.4%

COPD	163 (15.2)	9 (23.7)	.183	21.6%

Heart failure	91 (8.5)	6 (15.8)	.155	22.48%

Chronic renal failure	58 (5.4)	2 (5.3)	.965	-0.44%

Acute coronary syndrome	51 (4.8)	1 (2.6)	.507	-11.67%

Chronic liver failure	45 (4.2)	1 (2.6)	.609	-8.84%

Hematologic malignancy	12 (1.1)	0	.359	-14.91%

Solid tumor	124 (11.6)	4 (10.5)	.837	-3.51%

**Risk factors, number (%)**				

Urinary catheter	1,057 (99.1)	37 (97.4)	.387	-12.99%

Central venous catheter	1,044 (97.8)	38 (100.0)	.193	21.21%

Mechanical ventilation	967 (90.5)	37 (97.4)	.097	29.24%

Broad-spectrum antibiotics	941 (88.3)	36 (97.3)	.046	35.36%

Empiric/preemptive antifungal treatment.	180 (16.8)	2 (5.3)	.032	-37.31%

Arterial catheter	846 (79.5)	37 (97.4)	.001	58.33%

Enteral nutrition	825 (77.2)	26 (68.4)	.225	-19.87%

Total parenteral nutrition	428 (40.4)	27 (71.1)	< .001	64.99%

Corticosteroids	300 (28.5)	12 (31.6)	.679	6.76%

Renal replacement therapy	126 (12.0)	15 (39.5)	< .001	66.25%

^a^Median (25th to 75th percentiles). APACHE, Acute Physiology and Chronic Health Score; COPD, Chronic Obstructive Pulmonary Disease; SD, standard deviation; SDT, standardized difference; SOFA, Sequential Organ Failure Assessment.

The median duration from ICU admission to the onset of candidemia was 14 days (IQR 8.75 to 19.25 days). Abdominal surgery was the diagnosis on ICU admission in 31 (81.6%) patients. Infections were caused by *C. albicans *in 22 episodes, *C. parapsilosis *in nine, *C. tropicalis *in three, *C. glabrata *in two, *C. krusei *in one and *Candida ssp*. in one. The distribution of variables including severity indexes, ICU length of stay and mortality was similar in patients with candidemia caused by *C. albicans *and non-albicans *Candida spp*. As for the source of candidemia, infection of unknown origin was reported as the most frequent (*n *= 29, 76.3%) followed catheter-related infections (*n *= 8, 21.1%). One patient had candidemia and peritonitis concomitantly (2.6%). Multifocal colonization was a clinically relevant condition which was present in up to 91.4% of all patients before the development of candidemia. *C. parapsilosis *was the most frequently isolated species among the non-albicans spp in bloodstream infections and had a lower crude mortality (33%). Seven patients were not given antifungals and all of them died. Thirty-one patients were given antifungal treatment and in all cases the selection of the antifungal drug was appropriate according to the *Candida spp*. isolated. The following antifungal agents were used either as the sole agent throughout the course of treatment, or in a sequential pattern resulting in the use of multiple agents for a single episode (that is, de-escalation): fluconazole was used most often (*n *= 21, 67.7%), followed by amphotericin B-based (BL/CL) preparations (*n *= 7, 22.6%), caspofungin (*n *= 6, 19.4%) and voriconazole (*n *= 4, 12.9%). The median time for initiation of antifungal therapy was one day (IQR 0 to 4 days). In two patients with candidemia (5.7%), antifungal treatment was administered preemptively on the same day as material for blood culture was collected (day 0). The rest of the patients with candidemia were treated with targeted antifungal treatment after notification of a positive *Candida *blood culture result. Nearly one third of the episodes (*n *= 12, 31.6%) were treated between 24 and 48 hours (day one) after blood cultures were obtained (in some cases before final *Candida *species identification).

The crude ICU mortality rate was 52.6% (20/38) in patients with candidemia and 20.6% (220/1,069) in those without candidemia (*P *< 0.001). The crude hospital death rate was 55.3% (21/38) and 29.6% (314/1,069) among patients with and without candidemia, respectively (*P *= 0.01).

In the univariate analysis, age (hazard ratio (HR) 1.03, 95% CI 1.0 to 1.06; *P *= 0.032), APACHE II score on ICU admission (HR 1.09, 95% CI 1.12 to 1.81; *P *= 0.013), APACHE II score at diagnosis of candidemia (HR 1.13, 95% CI 1.03 to 1.24; *P *= 0.006), SOFA score at diagnosis of candidemia (HR 1.14, 95% CI 1.03 to 1.27; *P *= 0.011), and delay in starting antifungal therapy for more than 72 hours (HR 2.95, 95% CI 1.21 to 7.20; *P *= 0.017) were significantly associated with death. In the multivariate analysis, APACHE II score at the time of diagnosis of candidemia was the only predictor of death (adjusted HR 1.13, 95% CI 1.03 to 1.24; *P *= 0.006).

The logistic regression model used in propensity score analysis showed a high discriminatory power with an AUC value of 0.902. The standardized differences in the demographic and clinical variables of interest nearly disappeared when matched patients were analyzed (Table 2). In the matched study sample, the crude ICU mortality was 51.4% in the group with candidemia and 37.1% in the group without candidemia (*P *= 0.222). The crude hospital mortality rate was 54.3% among candidemic patients and 50% among non-candidemic patients (*P *= 0.680). After controlling residual confusion using a Poisson regression model, the risk of ICU candidemia-attributable mortality was relative risk (RR) 1.298 (95% CI 0.88 to 1.98) and the risk of hospital candidemia-attributable mortality was RR 1.096 (95% CI 0.68 to 1.69).

**Table 2:** Propensity score-matched patients with and without candidemia.

	Without candidemia (number = 70)	With candidemia (number = 35)	*P*	SDT
Age, years, mean (SD)	60.96 ± 17.3	59.2 ± 18.36	.641	9.86%

Male/female (%)	24 (34.3)	12 (34.3)	1	0%

APACHE II score at ICU admission, mean (SD)	19.07 ± 6.34	19.88 ± 6.29	.532	-12.82%

SOFA score at ICU admission	8 (4 to 10)	8 (7 to 10)	.658	

ICU length of stay (days)^*a*^	28.5 (17.75 to 40.25)	30 (17 to 58)	.514	

Hospital length of stay (dys)^*a *^	43 (28 to 69)	37 (27 to 70)	.801	

ICU mortality, number (%)	26 (37.1)	18 (51.4)	.163	

Overall mortality, number (%)	35 (50)	19 (54.3)	.680	

Diagnosis on ICU admission, number (%)			.861	

Medical	29 (41.4)	15 (42.9)		3%

Surgical	35 (50)	16 (45.7)		8.61%

Trauma	6 (8.6)	4 (11.4)		-9.34%

Multifocal colonization, total, number (%)	67 (95.7)	32 (91.4)	.398	-17.57

Clinical condition, number (%) at the time of ICU admission			.459	

No sepsis	6 (8.6)	1 (2.9)		-24.67

Sepsis	17 (24.3)	11 (31.4)		15.89

Severe sepsis	12 (17.1)	9 (25.7)		21.08

Septic shock	35 (50)	14 (40)		-20.2

Surgery, number (%)	35 (50)	16 (45.7)	.680	-8.6

Abdominal surgery, number (%)	26 (37.1)	11 (31.4)	.565	-12

Surgical procedures, number			.961	

None	34 (48.6)	18 (51.4)		5.6

One	19 (27.1)	9 (25.7)		-3.7

Two or more	17 (24.3)	8 (22.9)		-3.29

Secondary peritonitis, number (%)	9 (12.9)	3 (8.6)	.517	-13.9

**Underlying illnesses, number (%)**				

Neurologic disease	12 (17.1)	5 (14.3)	.709	-7.7

Trauma	9 (12.9)	6 (17.1)	.556	11.78

Diabetes mellitus	16 (22.9)	7 (20)	.739	-7

COPD	18 (25.7)	8 (22.9)	.749	-6.5

Heart failure	8 (11.4)	6 (17.1)	.419	16.36

Chronic renal failure	4 (5.7)	2 (5.7)	1	0

Acute coronary syndrome	1 (1.4)	1 (2.9)	1	0

Chronic liver failure	3 (4.3)	0	.549	-29.97

Hematologic malignancy	1 (1.4)	0	1	-16.85

Solid tumor	7 (10)	4 (11.4)	1	-4.5

**Risk factors, number (%)**				

Urinary catheter	70 (100)	34 (97.1)	.333	-24.4

Central venous catheter	70 (100)	35 (100)	-	0

Mechanical ventilation	69 (98.6)	35 (100)	1	16.8

Broad-spectrum antibiotics	69 (98.6)	34 (97.1)	1	-10.35

Empiric/preemptive antifungal treatment.	5 (7.1)	2 (5.7)	1	5.7

Arterial catheter	70 (100)	34 (97.1)	.333	-24.4

Enteral nutrition	49 (70)	25 (71.4)	.880	3

Total parenteral nutrition	55 (78.6)	25 (71.4)	.418	-16.78

Corticosteroids	31 (44.3)	12 (34.3)	.326	-20.58

Renal replacement therapy	24 (34.3)	14 (40)	.566	11.8

^a^Median (25th to 75th percentiles). APACHE, Acute Physiology and Chronic Health Score; COPD, Chronic Obstructive Pulmonary Disease; SD, standard deviation; SDT, standardized difference; SOFA, Sequential Organ Failure Assessment.

## Discussion

General findings obtained in this study, including an incidence rate of 34.3 episodes of candidemia per 1,000 ICU patients and a crude mortality rate of 52.6% are consistent with previously published data [5,20]. Attributable ICU mortality to *Candida*-related bloodstream infection varies largely and has been estimated to be non-significantly different from that of patients without candidemia or to be higher by 5% to 40% [7,21,22]. For this reason, analysis in matched cohort samples is recommended, although only seven studies assessing attributable mortality of candidemia have been previously published [23]. Important limitations are related to methodological heterogeneity, including study design (usually retrospective), study population (patients hospitalized in ICUs and hospital wards and those undergoing transplantation), source of data (hospital or microbiological databases) and matching criteria mainly based on underlying disease and comorbidity, rather than on all possible factors that may influence mortality. Moreover, severity scores and length of ICU stay were not considered.

In the present prospective and multicenter study, candidemia was not associated with an increase in either ICU or hospital mortality. This finding may be explained by the use of propensity matching score analysis to control for all potential confounding variables related to the development of *Candida *spp. bloodstream infection. Matching was highly effective and both candidemic and non-candidemic groups had similar characteristics, differing only in the development of bloodstream infection.

These favorable results could be attributed to earlier treatment of bloodstream infection and better monitoring (weekly surveillance sampling). An early start of appropriate antifungal therapy (within the critical time-frame of the first 24 to 48 hours) is crucial for the reduction of bloodstream infection-related mortality [24,25]. This observation confirms the importance of an empirical and preemptive strategy. Another important factor is the rapid reporting of the microbiological results (positive bloodstream infection and species identification) in order to initiate targeted antifungal treatment or to modify previous empirical antifungal agents. In our study, the median time for initiation of antifungal therapy was one day (IQR 0 to 4 days). In 36.9% of patients it was initiated within 48 hours of obtaining the first positive blood culture (5.2% of them as preemptive treatment). Another important problem preventing the earlier recognition and treatment of candidemia is the lack of specific clinical findings. Different prediction rules based on a variety of risk factors, including *Candida *species colonization are recommended to identify patients at high risk for fungal bloodstream infections [26,27]. High density colonization can be used to identify patients who may benefit from preemptive antifungal treatment in the appropriate clinical setting [28]. In our study multifocal colonization was a clinically relevant condition which was present in up to 92.1% of all patients before the development of candidemia and there was a high similarity between causative and colonized strains; this could guide appropriate antifungal treatment. Additionally, an appropriate antifungal agent was administered in all treated patients and this may also contribute to increased survival. Moreover, earlier replacement of central venous catheters, common practice in ICU's, could also reduce mortality. On the other hand, antifungal therapy was initiated in 180 non-candidemic high-risk patients (*n *= 180/1,069, 16.8%) as an empiric or preemptive strategy. This antifungal strategy has not been evaluated in previous articles estimating candidemia-attributable mortality. As this can be a major confounding factor, empirical or preemptive treatment was introduced in the logistic regression model. There was no difference in this variable in the propensity score-matched patients as we list in Table 2. It has been shown that ICU patients with candidemia have a substantially higher severity of illness on the day of diagnosis compared with the ICU admission day [29]. This is also an important disadvantage for matching because severity scores are determined on ICU admission but not at the time of developing candidemia. However, after matching by the propensity score, APACHE II and SOFA severity scores recorded weekly did not vary significantly between cases and controls.

The crude ICU mortality in patients with candidemia in our study is 52.6% with a median of five days from the diagnosis until death. Due to the high mortality and poor prognosis of patients with *Candida *spp. bloodstream infection, there is a strong need to identify predictors of death. Factors associated with fatal outcome, such as age, malnutrition, delay in removal of central venous catheter, candidemia caused by non-albicans *Candida *spp. and delay in starting antifungal therapy have been extensively recognized [25,30-32]. However, in our study only APACHEII score at the time of diagnosis of candidemia was a predictor of mortality.

The lack of differences in mortality rates between the groups of *C. albicans *and non-albicans *Candida *spp. may be explained by different reasons: i) Adequacy of antifungal therapy with all candidemias caused by *Candida *spp. with a possible decreased sensitivity or resistance to azoles being treated with candins or different formulations of amphotericin B; ii) a relatively high percentage of cases caused by *C. parapsilosis *(23.7%), associated with lower mortality [1,33,34]; and iii) a systematic removal policy for central venous catheters.

Two frequently cited studies [25,29] have shown an association between mortality and delay in start antifungal treatment. It should be noted that in both studies, the majority of episodes of candidemia occurred in patients admitted to hospital wards, with an in-hospital mortality rate of about 30%. In our study, all patients had ICU-acquired candidemia, with a higher crude mortality. It may be possible that antifungal treatment is started earlier in critically ill patients admitted to the ICU than in less severely ill patients in the hospital wards where strict protocols of microbiological surveillance are not established.

Despite the fact that more than 1,000 patients were recruited, the small number of candidemias related to the incidence of invasive candidiasis in the critically ill patient is a limitation of the study and might have compromised our power to detect differences in mortality between cases and controls. Additionally, the sensitivity of blood cultures for candidiasis is low (approximately 60%). This could impact the actual incidence of candidemia and also the overall mortality in the ICU. Therefore, there is a possibility that patients with candidemia that may have had identical matching risk factors but negative blood culture were considered matching controls instead of cases.

## Conclusions

In summary, ICU-acquired candidemia in critically ill patients was not associated with an increase of either ICU or hospital mortality. APACHE II at the time of diagnosis of candidemia was the only predictor of death in patients with candidemia.

## Key messages

• Candidemia was not associated with an increase in either ICU or hospital mortality.

• The use of propensity score matching analysis to control for all potential confounding variables allowed the assessment of candidemia-attributable mortality in critically ill patients.

• Earlier treatment of bloodstream infection and better monitoring (surveillance sampling weekly), resulting in appropriate antifungal agent may contribute to increased survival.

• APACHE II at the time of diagnosis of candidemia was the only predictor of death in patients with candidemia.

## Abbreviations

APACHE II: Acute Physiology and Chronic Health Evaluation; AUC: area under curve; CAVA: **Ca**ndida Score **Va**lidation Study Group; CFU: colony-forming unit; CI: confidence interval; COPD: chronic obstructive pulmonary disease; HR: hazard ratio; IQR: interquartile range; ROC: receiver operating characteristics; RR: relative risk; SD: standard deviation; SDT: standardized differences test; SOFA: Sequential Organ Failure Assessment; spp: species.

## Competing interests

The authors declare that they have no competing interests.

## Authors' contributions

FGdM was involved in data collection, contributed to writing of the manuscript and performed the statistical analysis. CL contributed to the conception and design of the study and revision of the manuscript. SRS contributed to the study design and revision of the manuscript. PS was involved in data management and data analysis. All authors read and approved the final version of the manuscript.
